# Reconstruction of the Image Metric of Periodic Structures in an Opto-Digital Angle Measurement System

**DOI:** 10.3390/s21134411

**Published:** 2021-06-27

**Authors:** Alexander N. Korolev, Alexander Ya. Lukin, Yurii V. Filatov, Vladimir Yu. Venediktov

**Affiliations:** 1 Laser Measurement and Navigation Systems Department, Electrotechnical University “LETI”, 197376 St. Petersburg, Russia; al_korolev@mail.ru (A.N.K.); yvfilatov@etu.ru (Y.V.F.); 2 Department of Physics, Peter the Great St. Petersburg Polytechnic University, 195251 St. Petersburg, Russia; alexander_lukin@inbox.ru; 3 Quantum Electronics Department, Faculty of Physics, Saint Petersburg State University, 198504 St. Petersburg, Russia

**Keywords:** goniometer, angle measurement, 2D mark

## Abstract

Measurement of the object angular position and its change is one of the important tasks in measurement technique. Our method is based on determination of the angular position of a 2D periodical optical pattern (2D mark) at the object, captured by the sensor of a digital camera. System performance can be frustrated by errors in determination of the spot coordinates on the camera sensor; by the presence of lens aberrations; by deviations from the parallelism of the pattern planes and the camera sensor; and by differences between the actual spots positions and the ideal grid. In the paper we discuss the effect of these errors and the way to correct or eliminate them. We have developed the mathematical routine and the corresponding numerical codes for correction of the said errors. The code and the routine we checked in a real experiment. It has shown that the correction decreases the standard deviation in 15 times.

## 1. Introduction

Angle measurement is one of the most ancient areas of metrology. The peculiarity of angle measurements is the fact that the angle, by definition, is a dimensionless quantity, representing a certain fraction of the total angle 2π, which, in turn, is the only obvious natural standard of the angle. As an angular measure, an optical polygon is most often used, the angles between the faces of which can be measured with an accuracy of about 0.1 arc-sec using a turntable and an autocollimator [1]. Nevertheless, the possibilities of using optical polygons in angular measurements are extremely limited due to the large value of the minimum angle between the faces (usually not less than 10 degrees). In this regard, in recent years in angular measurements, the use of such means as optical encoders [2,3] has become increasingly common. These angular measurement tools are based on circular scales and come in both incremental and absolute types. With the use of encoders on circular scales, the most accurate angular comparators were created, providing angular measurements with errors at the level of 0.01 arc-sec and better [4,5,6,7].

In circular scales, the distance between neighboring strokes of the scale divided by its radius determines the angular price of the scale division. A further increase in resolution is achieved by using various interpolation methods [8]. The distance between the strokes is almost impossible to make less than the wavelength of light due to diffraction restrictions. The smallest distance between the strokes is achieved by using a holographic approach when creating it and is on the order of the wavelength [9]. Further reduction of the division price is achieved only by increasing the radius of the circular scale. That is why in the best angle measuring systems (for example, the angle comparator, PTB, Germany [4]), the diameter of the angle scale reaches 400 mm. When using ring lasers (RL) [10,11], the circular scale is formed by the structure of the electromagnetic field of counter propagating waves, and the distance between the strokes is equivalent to the period of the standing wave formed in the RL resonator. This distance is also determined by the wavelength of the light. All this indicates that the known measurement technologies based on radial scales have reached some limits, and further improvement of their accuracy becomes more and more complex. So, if the limitation in diameter of the scale is on the level of 200 mm and the wavelength of light is about 0.5 μm, the resolution of the scale is limited by 1 arc-sec (without interpolation).

In [12], the authors proposed a new angle measurement technology based on the use of a 2D-pattern. The rotation angle measurement is based on measuring the rotation of the pattern image on the sensor of a digital camera. The article [12] formulated the main differences between the new concept of angle measurement and presented the first results of experimental and model studies of the metrological parameters of the new angle sensor, as well as mathematically proved the potentially extremely high accuracy of this method and the absence of its binding to the axis of rotation. The resolution of the proposed method (without interpolation) in this case is determined by the size of the camera pixel (3–4 μm) divided by the radius of the scale (20 mm) and the root of the number of 2D-pattern elements (100,000). For these values, we get 0.1 arc-sec. It is obvious that these values will improve with the development of technology. The inherent ability of this approach to generate angle standards in the form of digital files was also demonstrated. Later the authors have significantly improved the system performance [13,14].

During the research of the new angle sensor, the authors came to the conclusion that before starting metrological research and getting high accuracy, it is necessary to solve the problem of correcting image distortions in a real optical system.

These distortions are caused by

- the mutual inclination of the mark, the lens and the photodetector matrix,
- lens distortion,
- the manufacturing error of the mark.

Only after clearing the image of these distortions it is possible to start the next cycle of research and try to get high accuracy.

This article is devoted to the solution of this problem. 

## 2. 2D-Optical Pattern

The configuration of the optical pattern in the technology under consideration is a two-dimensional set of elements with a known location, namely, an orthogonal grid of elements in the form of ring with a relative brightness of 1 against a background with a relative brightness of 0. Since the orthogonal grid has the property of symmetry with respect to rotations at certain angles (0, 90, 180 and 270°), labels that uniquely determine the orientation of the pattern are needed. The pattern has three solid circular elements that form an isosceles triangle. The position of this triangle determines the orientation of the grid and provides a measurement range of 0–360°. Thus, we are talking about the representation of the angle scale in the form of a two-dimensional information field, which is reflected in the concept of “two-dimensional scale”.

The sensor of modern digital cameras is a two-dimensional array. The accuracy of sensor’s topology is tens of nanometers and is provided by modern integrated technologies. At the same time, the determining parameter is the size of the minimum element of the integrated circuit, which over the past 10 years in the process of technology development has constantly decreased from 65 to 22 nm [15]. The number of the elements of the image sensor can be millions or even tens of millions, with the dimensions of the elements being units of micrometers. Given the orthogonal topology and high accuracy of such structures, the effectiveness of their use for solving precision measurement problems is beyond doubt. From the metrological point of view, the image sensor is a unique device that simultaneously generates an information signal and is a two-dimensional measuring scale [15]. The violation of the sensor geometry is possible only due to the uneven heating and the associated thermal expansion.

**Figure 1:**
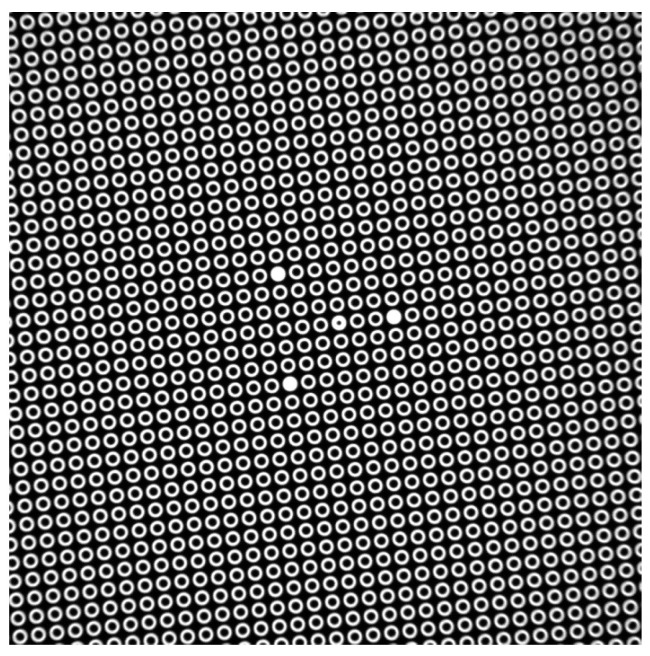
2D-optical pattern. The position of three emphasized elements provides the non-equivocal determination of the pattern orientation.

Figure 1 shows the image of the pattern, rotated at an angle of 7 deg, obtained using a digital camera with the sensor parameters 1280 × 1024 pixels, the pixel size is 5.2 × 5.2 μm; the pattern parameters − the diameter of the elements is 100 μm, the period is 150 μm; the lens magnification is –1^×^ (1^×^, 40 mm WD CompactTL™ Telecentric Lens, Edmund Optics product). The position of the 2D pattern perpendicular to the optical axis was adjusted using an autocollimator and structural elements.

To measure the angle from the digital image of the pattern, first the coordinates of all its elements in the sensor analysis area are determined as the position of the centers of the rings corresponding to each element of the pattern in relation to the coordinate system of the image sensor. Next, the rotation angle is calculated using the least square method.

Obviously, the larger the number of grid elements and the size of the digital camera image sensor, the higher the accuracy of the angle measurement. Theoretical analysis and modeling show that the error in measuring the angle Δ (in radians) depends on the error in determining the coordinates of individual elements Δs (in pixels) as:

$$\Delta \varphi =\frac{\Delta s}{R\sqrt{N}}$$

where *R* is the radius of the image analysis zone (in pixels), and *N* is the number of elements’ images in it.

## 3. Theory

Ideally, the image of the pattern on the camera sensor forms a rectangular grid of spots with a step *H*. Then the coordinates of the pattern elements rotated by an angle ϕ would be

(1) $$\begin{array}{l} x_{n}=iH\cos\varphi -jH\sin\varphi +b_{x}\\ y_{n}=iH\sin\varphi +jH\cos\varphi +b_{y} \end{array}$$

where *i*,*j*—numbers of a column and a row of the nth element the grid, $x_{n,\;}\;y_{n}$—its coordinates in the camera sensor with the origin of coordinates situated in the sensor center,   cosφ,  sinφ—elements of the rotation matrix, $b_{x},\;b_{y}$—the shift of the rotation axis from the spot (0,0).

Let us simplify the outlook of the equations. Let *a*_x_ = *H* cos *φ*, and *a*_y_ = *H* sin *φ*. Then:

(2) $$\begin{array}{l} x_{n}=a_{x}i-a_{y}j+b_{x}\\ y_{n}=a_{y}i+a_{x}j+b_{y}\\ n=1..N \end{array}$$

where *i*,*j*—numbers of a column and a row of the *n*-th element of the grid, $x_{n,\;}\;y_{n}$—its coordinates in the camera sensor with the origin of coordinates situated in the sensor center,   cosφ,  sinφ-elements of the rotation matrix, *b_x_, b_y_*—the shift of the rotation axis from the spot (0,0), $a_{x}=H\cos\varphi ,\;a_{y}=H\sin\varphi$.

For simplicity, we will assume the magnification factor in the optical system to be equal to 1^×^, so there is no difference between the step of the pattern and the step of its image. Equations (2) contain 4 unknown coefficients $a_{x},\;a_{y},\;b_{x},\;b_{y}$, therefore two spots are enough for coefficients determination. On the other hand, each pair of spots provides its own coefficient values due to errors in coefficient determination. A suitable approach in this case is to consider an overdetermined system of 2N equations of the form (2), where N is the number of spots, and its solution by the least squares method.

However, such a simple dependence is disrupted due to

(a) errors in determination of the coordinates of the spot on the camera sensor;
(b) the presence of lens aberrations;
(c) deviations from the parallelism of the planes of the pattern and the camera sensor;
(d) differences between the actual spots positions and the ideal grid.

The presence of lens aberrations leads to the appearance of additional shifts in the coordinates on the camera sensor, depending on the current position of the spot image $x_{n},\;y_{n}$. In this case, the most significant aberration is distortion. The classical expression for it contains terms of the 3rd and higher odd orders (to shorten the notation, we will restrict ourselves to 5). Written in a vector form, it contains two coefficients and center coordinates:

(3) $$\begin{array}{l} \Delta R=F_{3}RR^{2}+F_{5}RR^{4}\\ R=r-r_{0} \end{array}$$

Passing to the coordinate record, we have

(4) $$\begin{array}{l} \Delta x=(x-x_{0})\left(F_{3}+F_{5}\left({\left(x-x_{0}\right)}^{2}+{\left(y-y_{0}\right)}^{2}\right)\right)\left({\left(x-x_{0}\right)}^{2}+{\left(y-y_{0}\right)}^{2}\right)\\ \Delta y=(y-y_{0})\left(F_{3}+F_{5}\left({\left(x-x_{0}\right)}^{2}+{\left(y-y_{0}\right)}^{2}\right)\right)\left({\left(x-x_{0}\right)}^{2}+{\left(y-y_{0}\right)}^{2}\right) \end{array}$$

Distortion center coordinates $x_{0},\;y_{0}$ appear in the Equation (4) nonlinearly, complexifying the solution of the problem. Moreover, the aberrations are not required to have strict symmetry, so it is reasonable to generalize relation (4) to a polynomial with arbitrary coefficients. It is easy to see that the right side of Equation (4) contains products $x^{k}y^{m},\;\;k+m\leq 5$, i.e., the total degree of the factors does not exceed 5. Therefore, in generalized form the approximation for aberrations can be written as

(5) $$\begin{array}{l} \Delta x=\sum_{k=0}^{5}\sum_{m=0}^{5-k}F_{km}x^{k}y^{m}\\ \Delta y=\sum_{k=0}^{5}\sum_{m=0}^{5-k}G_{km}x^{k}y^{m} \end{array}$$

Each of the sums on the right side of (5) contains 21 coefficients; coefficients should be determined from the measurement results. In fact, the number of coefficients is less, since when (5) is added to (2), the constant terms $F_{00},\;G_{00}$ enter the system of equations in the same way as $b_{x},\;b_{y}$, making the system the degenerated one. In addition, the coefficients $F_{10},\;G_{01}$ reveal the specific behavior. Namely, in the case if the corresponding terms $F_{10}x,\;G_{01}y$ are transferred to the left side of (2), there takes place an arbitrarily change the scale of the variables, eventually turning the system into a homogeneous system with a trivial solution. Therefore, an additional condition to (5) is

(6) $$F_{00}=G_{00}=F_{10}=G_{01}=0$$

So, the corresponding coefficients are excluded from the system. System (2) with corrections (5) contains 4 + 2 × 19 = 42 coefficients, which can be found when the number of spots is 21 or more.

The non-parallelism of the planes of the pattern and the camera sensor leads to additional geometric distortions of the image. They can easily be calculated by combining the plane of the sensor in the reverse movement with the pattern plane.

**Figure 2:**
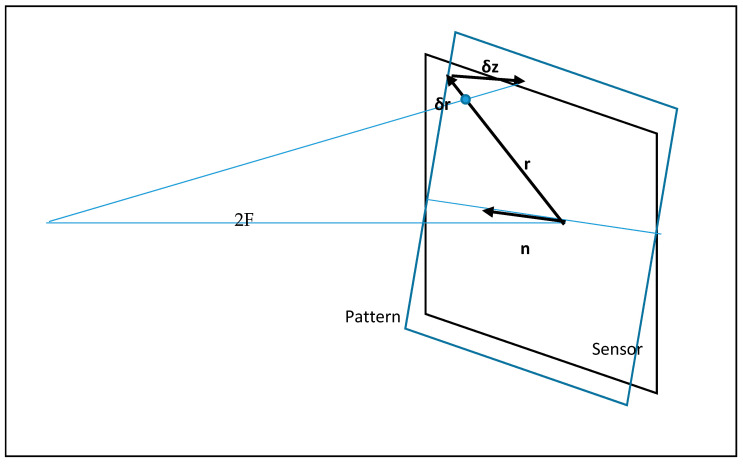
Combining the image sensor and the pattern in the reverse course.

Suppose that the camera sensor is perpendicular to the optical axis, and the pattern is tilted at a small angle α=αn, where n is the unit vector directed along the tilt axis (Figure 2). Obviously, n lies both in the plane of the sensor and the pattern, exactly on the line of their intersection.

When rotating at a small angle α, point displacement is calculated through cross product δz=[αr]=α[nr]. Vector δz is perpendicular to the plane K and parallel to the optical axis, thus   (r−δr)/2F=δr/δz. Considering the smallness of δr and its direction along r, we have

(7) $$\begin{array}{l} \delta r=-r\delta z/2F=-(ix+jy)\alpha (n_{x}y-n_{y}x)/2F\\ \delta x=\left(n_{y}x^{2}-n_{x}xy\right)\alpha /2F,\;\;\;\;\;\;\delta y=\left(n_{y}xy-n_{x}y^{2}\right)\alpha /2F\\ \delta x=fx^{2}+gxy,\;\;\;\;\;\;\delta y=fxy+gy^{2}\\ f=n_{y}\alpha /2F,\;\;\;\;g=-n_{x}\alpha /2F \end{array}$$

If the pattern is perpendicular to the optical axis, and the camera sensor is tilted, the constructions have a similar appearance. Therefore, if both the sensor and the pattern are tilted, the distortions are determined by the total angle between their planes. Thus, for considering the tilt, two coefficients f,g (6) are enough.

If we use expression (5) for aberrations compensation, then in the case of processing of one measurement, all the parameters of the tilt of the pattern and the sensor are already contained in (5).

Coordinates of ideal pattern elements can be written as $x_{n}=iH,\;\;y_{n}=jH$. In a real pattern coordinates will differ: $x_{n}=iH+\Delta x_{n},\;\;\;y_{ij}=jH+\Delta y_{n}$. Obviously, if we add unknown $\Delta x_{n},\;\;\Delta y_{n}$ to the system (2), it becomes incompletely determined – the number of equations turns out to be less than the number of unknown coefficients. Obviously, distortions caused by the aberrations and the pattern tilt are indistinguishable from the disruption of the regularity of the pattern. Therefore, the only way to separate them is to jointly process the data obtained at different angular positions of the pattern.

Each angular position has its own set of coefficients $a_{x},\;a_{y},\;b_{x},\;b_{y}$ and tilt correction coefficients (if the pattern tilt changes with turns), while the polynomial coefficients describing aberrations $F_{km},\;G_{km}$ and deviations $\Delta x_{n},\;\;\Delta y_{n}$ remain unchanged. Therefore, with the addition of $x_{n,\;}\;y_{n}$ for each new position, the number of equations increases by two times the number of spots, and the number of unknowns increases by 4 + (slope). When the number of spots is more than 23, two positions are sufficient for calculations; however, an increase of the number of turns can significantly increase the accuracy of approximation by reducing the influence of a random error in determining the coordinates of spots.

Since the coordinates of the pattern elements enter the system through their numbers i,j, it is convenient to introduce real element numbers, that differ from integers by a small correction

(8) $$\begin{array}{l} x_{n}=iH+\Delta x_{n}=\left(i+\frac{\Delta x_{n}}{H}\right)H=i_{R}H\\ y_{ij}=jH+\Delta y_{n}=\left(j+\frac{\Delta y_{n}}{H}\right)H=j_{R}H \end{array}$$

The transition to real numbers $i_{R},j_{R}$, however, does not solve another problem: the unknown $i_{R},j_{R}$ are multiplied by unknown coefficients $a_{x},\;a_{y}$ and the system of equations ceases to be linear. Therefore, an iteration method is used to solve it. In the initial approximation, integer numbers of the pattern elements are used, serving for calculation of the unknown coefficients of rotation, shifting, tilt corrections and aberrations. Using the obtained coefficients, deviations of each pattern element from the estimated position are calculated, and then they are averaged over all measured positions of the element (rotations of the pattern). Obtained deviation is used to calculate the real (corrected) numbers of the pattern elements. The calculations are repeated until the correction effect becomes less than a given value.

## 4. Experiment

This method has been implemented in the computer program and tested with the pattern and the camera described in the first section. When performing angle measurements, the program calculates the displacement of the pattern elements relative to the nodes of the above-mentioned ideal grid.

**Figure 3:**
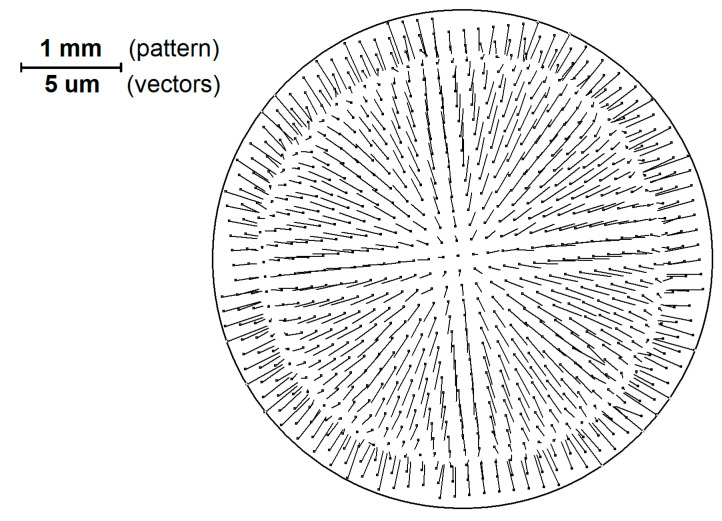
The structure of vectors that display the displacement of the elements of the pattern image relative to the ideal grid (enlarged 200 times compared to the scale of the pattern).

In Figure 3 the structure of vectors that represents the pattern image (Figure 1) elements shift relative to the ideal grid is shown.

The results of this measurement were as follows. The measured angle U = 276.2356178 deg; the number of pattern elements in the range of analysis area was 867; and the standard deviation for all field elements σ = 0.2190 pixel.

It should be noted that the scale of the vectors in Figure 3 differs from the scale of the pattern by 200 times.

**Figure 4:**
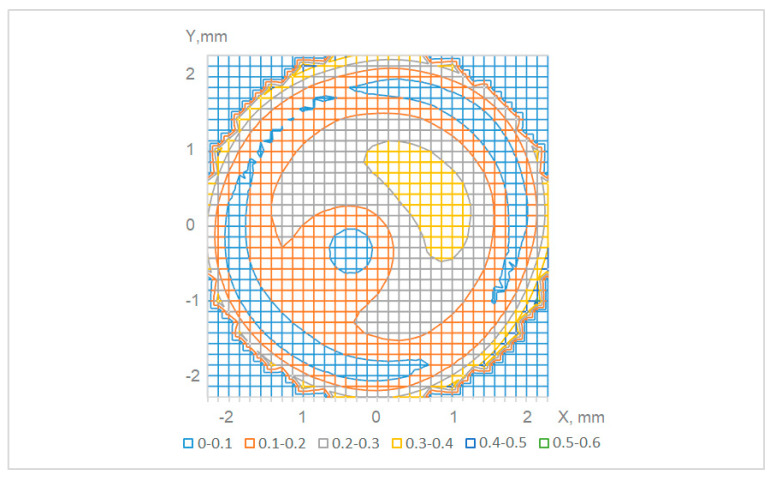
Modulus of shift vectors’ length for various points of the sensor. Different colors correspond to different ranges of length, measured in 1 tenth of the pixel size.

When measuring the angle, the coordinates of the centers of the pattern elements are calculated with an accuracy of hundredths of a pixel. In this case, an array of deviations of the elements’ centers along the *X* and *Y* axes relative to the ideal grid *dx (X, Y)* and *dy (X, Y)* is formed. Based on these shifts, a shift vectors field is possible to be constructed, representing the distortion and mutual inclination of the sensor pattern (Figure 4). The bottom row of color indices in Figure 4 shows the scale of shifts from 0 to 0.6 pixels with 0.1 pixel increment.

Thus, the task of this study is to divide image distortions of several micrometers into errors of the pattern itself, distortions from lens distortion and plane inclination errors, calculate their parameters and form the correction software that would ensure the restitution of the pattern image during measurements.

The basic concept of error separation is that pattern production errors are tied to the pattern element, and distortions are tied to the points of the image field.

Therefore, to identify all the errors, it is necessary to analyze series of pattern images.

The program provides automatic accumulation of series of images, their analysis and calculation of the parameters of the correction files.

The below mentioned results of analysis correspond to the processing of a series of 12 images, which are formed when the pattern is rotated every 30 deg.

When processing sequential analysis of the above-mentioned series of images, the obtained arrays of coordinates for all image elements of patterns *dx (X, Y*) and *dy (X, Y)* are added to an aggregate file, after which a two-dimensional approximation of the shift field by a 5th order polynomial is performed. Classical distortion is described by a third order polynomial. However, in modern lenses aspherics is used for distortion reduction; which makes it possible to change the direction of distortion at the periphery of the field of view, therefore, a 5th order polynomial is applied. This property of real distortion is also associated with a change in the direction of the shift vectors in Figure 3. At this stage, all the shifts associated with the inaccuracy of the pattern manufacturing can be considered as white noise that does not seriously affect the calculation of the global distortion function.

**Figure 5:**
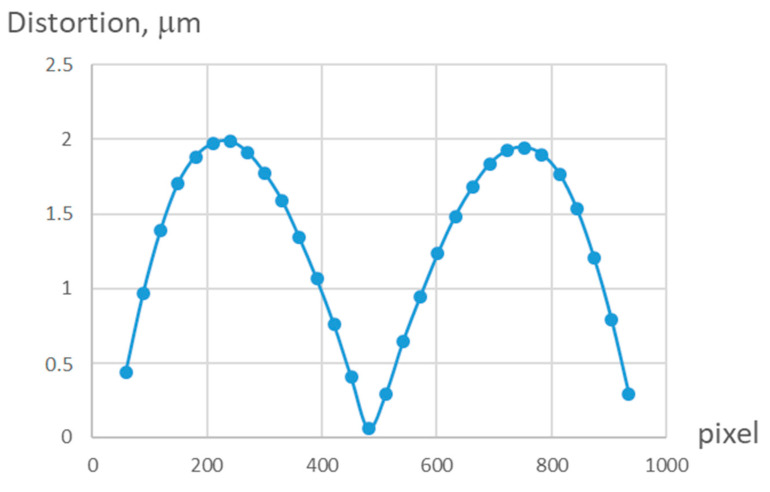
Plot of the central section of the two-dimensional distortion function of the 5th order, calculated from a series of 12 frames.

Figure 5 shows a graph of the central section of a 5th order two-dimensional distortion function, calculated from a series of 12 images. As can be seen from the graph, the depth of distortions does not exceed 2 μm.

Based on the calculation results, the distortion correction data is generated. This data contains coefficients of all the terms of the polynomial, which is an analytical description of the distortion and is used later for its correction.

Further, the distortion is corrected sequentially for the entire series of images using this data. Now the vectors of shifts *dx (X, Y)* and *dY (X, Y)* for each image are free from distortion and include only their own displacements from ideal position in the form of deviations for each element. Then, to improve the accuracy, the indicated displacements of the elements are averaged over all images. The obtained average values are the basis for the formation of a pattern displacements correction data, having a form of an array of shifts by element numbers.

**Figure 6:**
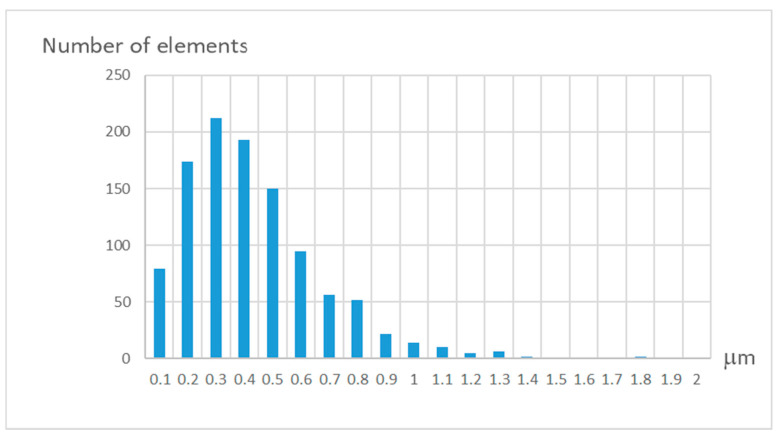
Histogram of pattern elements’ displacement.

In Figure 6 a histogram of the pattern elements’ displacement calculated in accordance with the above-mentioned procedure is shown. The range of deviations is within 1 μm. The error distribution is close enough to the normal law for the absolute deviation.

**Figure 7:**
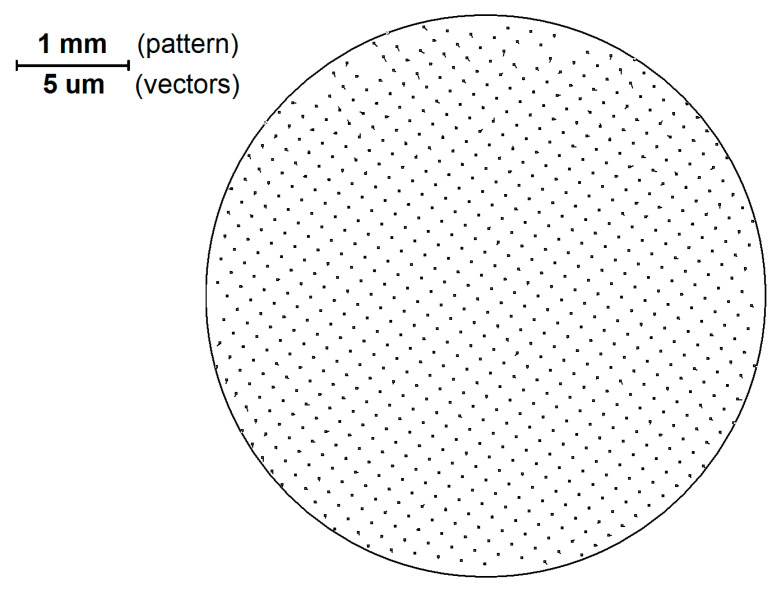
Image of the vector field after performing the correction using distortion and pattern displacements correction data (enlarged 200 times compared to the scale of the pattern).

In Figure 7 the image of the vector field after performing the distortion and pattern displacements correction is represented. Here the scale of the vectors is also 200 times in relation to the scale of the pattern, and vectors are almost invisible.

The use of correction files leads to a significant reduction of errors in the periodic structure of the pattern image.

The result of the measurements after correction are as follows. The measured angle U = 276.2355187 deg; the number of pattern elements in the range of analysis area was 867; and the standard deviation for all field elements σ = 0.0138526 pixel.

Comparison of the results shows that the correction decreases the standard deviation in 15 times.

## 5. Conclusions

The technology for the analysis and processing of images has been developed. It makes possible to restore the image metrics of periodic structures in the range of small values, that are a fraction of a image sensor pixel. This technology is the basis for further improving the metrological characteristics of goniometers with small-scale diameters, based on the measurement of image rotation.

## Data Availability

Not applicable.
